# Assessing the Market Readiness for Medical Cannabis in Greece: A Qualitative Study of Patient Perspectives

**DOI:** 10.3390/medicines12020012

**Published:** 2025-05-09

**Authors:** Christos Ntais, Yioula Melanthiou

**Affiliations:** 1 Healthcare Management Program, School of Economics & Management, Open University of Cyprus, 2220 Nicosia, Cyprus; 2 Marketing Department, School of Business, University of Nicosia, 2417 Nicosia, Cyprus; 3 Department of Communication and Marketing, Faculty of Communication and Media Studies, Cyprus University of Technology, 3036 Limassol, Cyprus; yioula.melanthiou@cut.ac.cy

**Keywords:** medical cannabis, clinical evidence, social stigma, regulatory framework, qualitative research

## Abstract

**Background:** The introduction of medical cannabis in Greece marks a shift in healthcare policy, yet patient attitudes remain underexplored. **Methods:** This qualitative study examines the market readiness for medical cannabis through semi-structured interviews with 24 participants—12 users of cannabidiol (CBD)-based formulations and 12 medical cannabis-naive individuals. **Results:** CBD-experienced patients generally perceive cannabis-based treatments as beneficial for managing musculoskeletal pain, migraines, anxiety, stress and sleep disturbances, despite concerns over product quality, cost and limited medical guidance. Medical cannabis-naive participants express skepticism due to stigma and perceived insufficient evidence but acknowledge potential therapeutic value within a regulated framework. This study highlights the need for better patient education, physician training and clear regulatory guidelines to support responsible market entry. **Conclusions:** These findings offer important insights for policymakers, healthcare providers and the pharmaceutical industry, emphasizing the need for a structured, evidence-based approach to medical cannabis integration in Greece. Further research is needed to assess long-term patient experiences and the evolving impact of regulatory changes on market dynamics.

## 1. Introduction

The potential benefits of medical cannabis are gaining attention, particularly in the management of conditions such as childhood epilepsy, spasticity in multiple sclerosis, chemotherapy-induced nausea and vomiting, chronic non-cancer pain and other disorders [1,2,3,4,5]. Over the past decade, numerous countries have adopted policies that facilitate patient access to cannabis-based therapies, reflecting growing acceptance of its medical potential [6,7]. Despite these advancements, significant challenges remain in integrating medical cannabis into mainstream healthcare, particularly in regions where its use has been historically stigmatized.

Greece has recently taken legislative steps toward regulating medical cannabis with the aim of creating a controlled framework for its cultivation, distribution and prescription. The legal status of medical cannabis varies depending on whether the formulation contains THC (tetrahydrocannabinol) or CBD (cannabidiol). The pharmacological activities of THC and CBD differ significantly, contributing to their distinct regulatory classifications. THC is the principal psychoactive component of cannabis, responsible for producing euphoria, altered perception and other central nervous system effects. Its psychoactive properties raise concerns about dependence, cognitive impairment and potential misuse, which necessitate stricter regulatory controls. In contrast, CBD is non-psychoactive and exhibits a broad range of pharmacological effects, including anti-inflammatory, anxiolytic, anticonvulsant and analgesic properties [1]. Because of its safer pharmacological profile and lack of intoxicating effects, CBD-containing products are subject to fewer legal restrictions than THC-based formulations. CBD-based treatments typically refer to products that contain primarily CBD, with minimal or legally negligible amounts of THC—usually less than 0.2% as per Greek regulations. These products, which include CBD oils, capsules and topicals, are often marketed as wellness supplements and can be purchased without a prescription in Greece. In contrast, full-spectrum medical cannabis encompasses products derived from the entire cannabis plant, containing a broader range of cannabinoids, including significant concentrations of THC. Full-spectrum medical cannabis is strictly regulated in Greece, available only through prescription and subject to bureaucratic control mechanisms. Thus, while CBD-based products are more easily accessible and perceived as safer by the general public, full-spectrum medical cannabis involves a more complex therapeutic profile, stricter legal controls and distinct clinical applications.

The actual implementation and acceptance of medical cannabis within the Greek healthcare system remain uncertain. Its market entry depends not only on regulatory clarity but also on the perceptions and readiness of key stakeholders, including patients, healthcare professionals and policymakers. This study investigates the perspectives of Greek patients regarding medical cannabis through semi-structured interviews with both users of CBD formulations and medical cannabis-naive individuals. By exploring patient experiences, expectations and concerns, this research aims to identify key facilitators and barriers to the responsible adoption of medical cannabis in Greece, to provide critical insights for policymakers, healthcare professionals and pharmaceutical industry stakeholders and to offer recommendations towards an evidence-based and patient-centered approach to integrating medical cannabis into the national healthcare framework.

## 2. Methods

### 2.1. Study Design

A qualitative research design was employed to explore patient perspectives on medical cannabis. Semi-structured interviews were conducted to elicit in-depth insights, allowing participants to elaborate on their experiences and viewpoints. The study adopted an inductive approach, employing content analysis to identify recurring patterns and themes.

### 2.2. Participants

Participants were recruited using a non-random convenience sampling approach from private pharmacies in Athens between November 2022 and April 2023. Inclusion criteria included the following: (i) being an adult aged 18 years or older, (ii) holding a university degree and (iii) either prior experience with CBD-based treatments or no prior use of cannabis-based products. Participants were required to hold a university degree to ensure that they could engage meaningfully in detailed discussions regarding medical cannabis, healthcare regulation and pharmaceutical policy—topics that often involve technical, legal and scientific aspects. This requirement aimed to promote a high level of comprehension and articulation during interviews, thereby enhancing the richness and reliability of the qualitative data. While this approach may limit generalizability to the broader population, it was considered appropriate for the study’s aim of gaining in-depth insights into informed patient perspectives on the market readiness for medical cannabis in Greece.

Potential participants were identified through collaboration with licensed private pharmacies in Athens, Greece. Pharmacists were briefed on the study’s eligibility criteria and asked to inform eligible customers about the opportunity to participate. Interested individuals were then provided with an information sheet describing the study’s aims, procedures and voluntary nature. Those who expressed interest were contacted directly by the research team, ensuring that pharmacists were not involved in participant selection. A total of 24 participants were enrolled, divided equally between CBD users (n = 12) and medical cannabis-naive individuals (n = 12). The CBD-experienced group consisted of individuals who had independently purchased CBD-based products over the counter from pharmacies, without a physician’s prescription or formal medical guidance. None of the participants in either group had prior experience with full-spectrum medical cannabis (containing significant levels of THC) obtained through legal or illicit channels. Recruitment efforts were spread across multiple pharmacy locations to enhance variability in demographics and health conditions among participants.

The determination of the sample size (n = 24) was guided by the principles of information power, as outlined by Malterud et al. [8]. According to this framework, the more information each participant holds relative to the study aim, the fewer participants are needed. Our relatively narrow study aim (exploring patient perspectives on the market readiness for medical cannabis in Greece), the application of a strong interview guide and the use of in-depth analysis techniques collectively supported a sample size on the smaller end of the qualitative research spectrum. Furthermore, thematic saturation was observed, as no new themes emerged in the final interviews, indicating that additional interviews were unlikely to yield substantially new information. This approach ensured the sample was sufficient to achieve the study’s objectives while maintaining methodological rigor.

### 2.3. Data Collection

Semi-structured, face-to-face interviews were conducted in private rooms at participating pharmacies or neutral venues agreed upon by the participants to ensure confidentiality and comfort. A flexible semi-structured interview guide was used to ensure consistency while allowing for follow-up questions tailored to individual responses (Table 1). Key topics included perceptions of medical cannabis as a therapeutic option, need for further scientific research, social stigma surrounding medical cannabis use and barriers to its adoption. Each interview lasted approximately 45 to 60 min and was audio-recorded with participants’ informed consent. Interviewees were informed of their rights, including voluntary participation and the option to withdraw at any point.

### 2.4. Data Analysis

Interview recordings were transcribed verbatim. Data were analyzed using thematic analysis following the six-step framework proposed by Braun and Clarke [9]. Initial coding was performed independently by the researchers to identify key patterns and emerging themes across participant responses. Codes were iteratively refined into broader themes through discussion among the research team to ensure consistency and reduce individual bias. NVivo 14 software was used to assist with data organization and analysis. Discrepancies in coding were resolved through consensus.

### 2.5. Ethical Considerations

Ethical approval for this study was obtained from the University of Nicosia Research Ethics Committee (Ref. No UREC/2022/14 dated 19 July 2022). Participants provided informed consent before taking part in the study, ensuring they understood the purpose of the research and their rights, including the right to withdraw at any time. Anonymity and confidentiality were maintained throughout data collection, analysis and reporting. To protect participants’ identities, participants were assigned aliases (“User 1”–“User 12” for CBD users; “Non-user 1”–“Non-user 12” for non-users). Data were securely stored and accessible only to the research team.

## 3. Results

### 3.1. Sample Characteristics

The study included 24 participants, equally divided into two groups: users (n = 12), who had prior experience with CBD-based treatments, and non-users (n = 12), who had never used medical cannabis. Users ranged in age from 24 to 52 years old, with a mean age of 38 years. Non-users ranged in age from 26 to 57 years old, with a mean age of 40 years. The sample was diverse in terms of gender (14 females, 10 males, equally divided within the two groups) and included individuals with various health conditions, such as musculoskeletal pain (n = 5), migraine (n = 6), anxiety (n = 5), stress disorders (n = 3) and sleep disturbances (n = 5). All participants were university graduates and residents of Athens, Greece. Table 2 summarizes the demographic characteristics of both CBD-experienced and medical cannabis-naive participants, including age, gender, health conditions, educational background and employment status.

### 3.2. Thematic Axes

#### 3.2.1. Perceptions of Medical Cannabis as a Therapeutic Product

Participants expressed varying views on the legitimacy of medical cannabis as a therapeutic alternative. CBD-experienced patients generally perceived cannabis-based treatments as beneficial, particularly for managing chronic pain, anxiety and insomnia. One user noted, “Cannabis oils have helped me manage my chronic back pain better than traditional medications” (User 3). However, concerns about inconsistent product quality and lack of standardized dosages were raised: “I never know if another brand with the same CBD content will have the same effect, and that’s frustrating” (User 7).

Other users praised CBD-based products’ efficacy for conditions where conventional medications had failed, with one stating, “I had tried so many different treatments, but nothing worked as well as cannabis for my musculoskeletal pain” (User 6). Another described how medical cannabis improved their overall well-being: “It doesn’t just help with my physical symptoms; I feel more relaxed and in control of my condition” (User 4).

Conversely, many cannabis-naive patients remained skeptical. One non-user stated, “Medical cannabis has rapidly and unexpectedly emerged as an alternative solution for serious diseases” (Non-user 1). Others viewed medical cannabis as underdeveloped: “It is not widely used, partly because of legislation issues and its questionable therapeutic status” (Non-user 10). Some non-users expressed concern about potential side effects, with one participant stating, “I worry about dependency and how long-term use could affect my cognitive function” (Non-user 8).

A few participants suggested that cannabis-based treatments should be more readily available but closely monitored. One stated, “If it helps people and is safe, then why not? But it should be regulated like any other medication” (Non-user 5). Another mentioned, “The government should focus on strict monitoring so that we can be sure about its long-term effects” (Non-user 2).

#### 3.2.2. Need for Further Scientific Research

A strong consensus emerged regarding the necessity for more rigorous scientific research. CBD users supported further research but acknowledged positive personal experiences, while non-users emphasized the need for clinical validation. One participant noted, “Extensive scientific research is a precondition for predicting cannabis’ medical use” (Non-user 4). Another emphasized, “More research should be encouraged as with most other natural remedies. Scientific evidence is necessary for both patients and doctors” (Non-user 5).

Several participants also highlighted the need for long-term studies to assess the effects of medical cannabis use. One user stated, “We need studies that go beyond short-term benefits and examine long-term safety and efficacy” (User 10). Another participant echoed this sentiment, emphasizing the importance of comparative studies: “How does cannabis compare with other treatments in terms of effectiveness and side effects over time?” (Non-user 6).

Participants also stressed the role of governmental and institutional support in facilitating high-quality research. One noted, “Without proper funding and backing, scientific studies on cannabis will remain limited” (User 9). Another added, “Collaboration between universities, public health authorities and the pharmaceutical industry is essential for credible research” (Non-user 3).

Furthermore, some participants advocated for more patient-centered research, emphasizing the need for studies that reflect real-world experiences: “Studies should include diverse patient groups to understand how cannabis works for different conditions” (User 8). Another remarked, “We need research that takes patient feedback into account, not just lab results” (Non-user 7).

#### 3.2.3. Stigma and Public Perception

Many participants cited stigma as a barrier to medical cannabis acceptance. One respondent explained, “Cannabis has been vastly connected with light drug use. For people to accept its medical use, it must be strongly and undisputedly proven” (Non-user 2). Even users faced societal judgment: “I sometimes hesitate to tell people I use CBD because of the stigma attached to medical cannabis” (User 8).

Several participants indicated that the portrayal of cannabis in the media contributes to negative public perceptions. One participant noted, “Most people still see cannabis as a drug rather than as medicine, and that mindset needs to change through proper education and awareness campaigns” (Non-user 4). Another added, “There needs to be more success stories in the media that highlight how medical cannabis has helped people recover or improve their conditions” (User 7).

Furthermore, some participants emphasized the role of healthcare professionals in reducing stigma. A non-user remarked, “If doctors themselves are hesitant to recommend it, how can patients feel confident in using it?” (Non-user 6). A user highlighted the need for physician training: “Doctors need more knowledge about cannabis so they can present it as a legitimate treatment rather than something controversial” (User 5).

The need for public education was also repeatedly mentioned. One participant stated, “If people were better informed about how cannabis works medically, they might be less judgmental” (Non-user 3). Another suggested, “Public discussions should be organized to change people’s attitudes and explain the science behind medical cannabis use” (User 9).

#### 3.2.4. Barriers to Widespread Use

Regulatory challenges, physicians’ hesitancy and cost were identified as key barriers. Some users expressed frustration over affordability: “CBD is too expensive and health insurance doesn’t cover it” (User 5). Another recurring concern was the lack of a strict regulatory framework for CBD products, leading to variations in quality and standardization. Some participants were uncertain about whether they could trust the available CBD products. One participant mentioned, “Since CBD formulations are not considered medicinal products in Greece, there’s no guarantee that what I’m getting is of pharmaceutical quality. We need better quality control and regulatory oversight” (User 7). Others echoed similar concerns, stating that inconsistent formulations made it difficult to rely on CBD-based treatments: “One brand works great, another doesn’t. This lack of standardization is a huge problem” (User 10).

Physician reluctance to recommend medical cannabis was also a major barrier identified by participants. Many reported encountering doctors who were unwilling to recommend medical cannabis due to a lack of knowledge or comfort with cannabis-based treatments. One participant remarked, “My doctor was completely against it, even when I explained that I had exhausted all other options” (User 8). Others emphasized the lack of physician knowledge: “Doctors need proper training on cannabis, otherwise patients will keep acting on their own” (User 12). Another added, “Legalization must be accompanied by comprehensive physician education to address their concerns and misconceptions” (Non-user 6).

Despite these barriers, some participants expressed optimism about the future. One participant stated, “If more doctors get on board and regulations improve, medical cannabis could really help a lot of people” (User 2). Another suggested, “Clearer policies could go a long way in making medical cannabis more accessible to those who need it” (Non-user 3).

## 4. Discussion

The findings of this study highlight several key areas that must be addressed to facilitate the responsible market entry of medical cannabis in Greece. First, regulatory frameworks need refinement to ensure smoother accessibility for patients who need cannabis for medical use. Prior research highlights that overly complex regulatory environments can hinder patient access to medical cannabis, as seen in other European jurisdictions [10]. The bureaucratic hurdles associated with obtaining THC products have created significant access barriers. Literature suggests that streamlining approval processes, reducing administrative burdens on healthcare professionals and improving clarity in legal provisions can enhance patient access while maintaining regulatory oversight [11]. Policymakers should consider adopting best practices from countries with more established medical cannabis markets, such as Germany and Canada, where patient-centered reforms have led to improved availability and safer consumption practices [12,13,14].

Another important aspect is the need for medical education and physician engagement. Many participants reported encountering resistance from doctors due to a lack of knowledge about medical cannabis. A survey of the Society of Cannabis Clinicians revealed that only 1 out of 45 physician respondents had received education about cannabis during medical school [15]. Consequently, many physicians sought knowledge through conferences (71%), medical literature (64%) and online resources (62%). Despite these efforts, just over half felt adequately informed to include cannabis in their clinical practice. Similarly, in a study assessing healthcare practitioners’ perceptions on medical cannabis, respondents expressed discomfort with their level of knowledge, despite over 80% having patients who use cannabis for medical purposes [16]. The study concluded that medical training programs must reassess their curricula to equip healthcare practitioners with the necessary knowledge and confidence to meet evolving patient needs. Furthermore, a scoping review found that allied healthcare trainees lacked sufficient knowledge about medical cannabis and did not feel prepared to counsel patients on the subject [17]. Both students and faculty expressed a desire for standardized education on medical cannabis, suggesting that implementing competency-based curricula is essential for preparing future healthcare professionals. In addition, evidence-based medical education related to cannabis has been called for in a recent position paper from the American College of Physicians [18]. These findings suggest that healthcare professionals require comprehensive training on cannabis-based treatments, including dosage guidelines, therapeutic benefits and potential risks. Implementing educational initiatives within medical schools and continuing education programs could help bridge the existing knowledge gap and enhance physician confidence in prescribing cannabis-based therapies.

Additionally, public attitudes and stigma remain significant obstacles. Despite legislative progress, medical cannabis continues to be perceived negatively by segments of society, including healthcare providers. A survey of primary care providers in Minnesota revealed that while 58.1% viewed medical cannabis as a legitimate therapy, only 38.7% believed it should be offered to patients [19]. Similarly, a study examining the intentions of healthcare providers to recommend medical cannabis found that negative attitudes and perceived stigma towards users were associated with a lower likelihood of recommending such treatments [20]. The study emphasized that stigma mediated the relationship between providers’ attitudes and their intention to recommend medical cannabis, suggesting that addressing stigma could enhance provider engagement. Public education campaigns and awareness programs could help dispel misconceptions and promote evidence-based understanding of cannabis as a legitimate medical treatment [21,22].

In the Greek context, historical and societal factors further reinforce stigma. Cannabis has traditionally been associated with illicit drug use, shaped by strict anti-drug policies and negative media portrayals during the late 20th century. This legacy contributes to persistent public skepticism, even regarding medical use. Additionally, Greek society tends to place considerable trust in formal medical authorities; thus, the limited guidance from healthcare professionals regarding medical cannabis exacerbates hesitancy. These cultural dynamics underline the importance of implementing not only physician training but also broader public education efforts to foster acceptance and responsible integration of medical cannabis into healthcare practice.

Comparative experiences from other countries provide useful insights into the challenges Greece faces in integrating medical cannabis into healthcare practice. For instance, in Germany, although medical cannabis was legalized in 2017 and access was facilitated through public insurance, both stigma and physician hesitancy persisted in the early years of implementation [12]. Similarly, in Canada, despite a long history of medical cannabis access, patients have reported ongoing difficulties with healthcare provider support and social acceptance [14]. These international experiences suggest that legalization alone does not eliminate stigma or ensure seamless clinical adoption. Greece’s efforts could benefit from lessons learned abroad, emphasizing the need for physician training, public education and streamlined regulatory processes alongside legalization.

Our study also underscores the importance of quality control and product standardization. Participants raised concerns about inconsistencies in CBD formulations, which made it difficult for patients to achieve reliable therapeutic outcomes. A study by Bonn-Miller et al. [23] analyzed 84 CBD products from 31 companies and found that 26% contained less CBD than labeled, while 43% contained more. Additionally, some products contained detectable levels of THC. Similarly, a study by Pavlovic et al. [24] demonstrated that the majority of commercially available CBD products tested were inaccurately labeled. Establishing strict quality assurance protocols, including lab testing and standardized production practices, could enhance patient confidence and ensure the safety and efficacy of CBD products.

This research is subject to some limitations. First, given the use of a convenience sample, the findings are not intended to be representative of the broader Greek patient population. Another limitation is the potential for response bias, as participants who chose to engage in the study may have stronger opinions—either in favor of or against medical cannabis—than the general population. Third, while the sample size was relatively small, practical research suggests that a sample of 12 participants may be sufficient to reach data saturation within a relatively homogeneous population [25]. However, a larger sample could have provided additional nuances.

Another limitation of this study is that it exclusively included medical cannabis users who receive CBD-based formulations, as opposed to THC-containing products. This restriction is due to the limited availability and accessibility of THC-based treatments in Greece, making it challenging to identify and enroll patients using such products. As a result, the findings may not fully capture the perspectives and experiences of individuals who rely on THC-inclusive treatments. Additionally, this study relied exclusively on qualitative data, which, while offering rich insights into patient perceptions, limits the generalizability of findings. Future research incorporating quantitative or mixed-methods designs could provide broader validation and enable statistical analysis of attitudes and trends within the larger Greek patient population. Finally, the study focused on patient perspectives, without incorporating insights from healthcare professionals, policymakers or pharmaceutical industry stakeholders, whose views are also critical in shaping the market landscape for medical cannabis.

## 5. Recommendations for Future Research

Future research should adopt a multidisciplinary approach to further explore the market readiness for medical cannabis in Greece. Longitudinal studies tracking patient experiences over time could provide deeper insights into the long-term benefits and potential risks of cannabis-based treatments. Additionally, expanding research to engage a more diverse participant pool, including policymakers, healthcare professionals and industry groups, would help capture a more comprehensive perspective on the challenges and opportunities associated with the responsible acceptance of medical cannabis in clinical practice. Future research should also aim to include a broader range of medical cannabis users, including those receiving THC-containing formulations, to provide a more comprehensive understanding of patient perspectives and experiences.

In addition, comparative studies examining the implementation of medical cannabis programs in other countries could provide valuable insights into best practices and potential pitfalls. Finally, studies on the social and economic impact of medical cannabis legalization in Greece could provide further insights into how public health, employment opportunities and economic growth are affected.

## 6. Conclusions

The responsible market entry of medical cannabis in Greece depends on addressing public concerns, regulatory clarity and scientific validation. While some patients acknowledge its therapeutic potential, overcoming stigma and misinformation remains a crucial step. Public awareness campaigns should be implemented to challenge societal misconceptions and destigmatize the use of medical cannabis within the healthcare system. Addressing stigma through targeted education and transparent policymaking will be key in achieving broader acceptance among both healthcare professionals and the general public.

Additionally, policymakers should consider revising regulatory frameworks to facilitate easier access while maintaining strict quality control measures. Collaboration between healthcare providers, policymakers and industry stakeholders will be vital in creating an evidence-based and patient-centered medical cannabis framework. By fostering interdisciplinary dialogue, Greece can develop a robust regulatory model that ensures safety, efficacy and equitable access. In conclusion, while challenges remain, a research-driven and patient-focused approach will help develop a structured national medical cannabis strategy.

## Figures and Tables

**Table 1:** Topic Guide.

**Section**	**Questions**
Introduction	- Thank you for participating in this study. The purpose of this interview is to explore your perspectives on medical cannabis and its readiness for market entry in Greece. - Your responses will be confidential, and you are free to withdraw at any time. - There are no right or wrong answers; we are interested in your honest opinions and experiences.
Background Information	Can you tell me a little about yourself (age, place of residence, educational background, health status)? Have you ever used any form of cannabis, including CBD products, for medical purposes?
Knowledge and Perceptions of Medical Cannabis	3. What do you know about medical cannabis and its use for health conditions? 4. Where do you get most of your information about medical cannabis (e.g., doctors, media, internet, social circles)? 5. Do you perceive medical cannabis as a legitimate treatment option? Why or why not?
Experiences with Medical Cannabis (For Users)	6. How did you first learn about medical cannabis as a treatment option? 7. What condition are you using it for, and how has it affected your symptoms? 8. What challenges, if any, have you faced in accessing medical cannabis in Greece? 9. How do you feel about the quality and consistency of available medical cannabis products? 10. What role has your doctor played in guiding your medical cannabis use? Have they been supportive or hesitant?
Barriers and Concerns (For Non-Users and Users)	11. What are the main concerns you have about medical cannabis? 12. Do you think stigma affects patients’ willingness to try medical cannabis? Why or why not? 13. Have you experienced or witnessed any social stigma associated with medical cannabis use? 14. In your opinion, what are the biggest obstacles to medical cannabis being included in Greece’s healthcare system?
Regulatory and Market Readiness	15. What are your thoughts on the current legal and regulatory framework for medical cannabis in Greece? 16. Do you think doctors are adequately trained to prescribe medical cannabis? 17. How do you feel about the availability and accessibility of medical cannabis in Greece? 18. What changes would you like to see in policies and regulations regarding medical cannabis?
Future Considerations and Recommendations	19. What would make you (or more patients) feel more comfortable using medical cannabis? 20. What role should the government and medical community play in educating people about medical cannabis? 21. What recommendations would you give to policymakers to improve the accessibility and acceptance of medical cannabis in Greece?
Closing Remarks	- Thank you for sharing your thoughts and experiences. - Is there anything else you would like to add that we haven’t covered? - Your insights are valuable and will contribute to a better understanding of how medical cannabis can be responsibly integrated into the healthcare system in Greece.

**Table 2:** Demographic characteristics of the study participants (n = 24).

	CBD Users (n = 12)	Non-Users (n = 12)	Total (n = 24)
Age (mean, range)	38 years (24–52)	40 years (26–57)	39 years (24–57)
Gender			
- Female	7	7	14
- Male	5	5	10
Health Conditions			
- Musculoskeletal Pain	3	2	5
- Migraine	3	3	6
- Anxiety	2	3	5
- Stress Disorders	2	1	3
- Sleep Disturbances	2	3	5
Educational Level			
- University Degree	12	12	24
Employment Status			
- Employed	10	10	20
- Unemployed/Student	2	2	4

## Data Availability

The data that support the findings of this study are available from the corresponding author upon reasonable request.
